# Spontaneous regression of hepatocellular carcinoma after alcohol and smoking abstinence and bone fracture surgery: A case report

**DOI:** 10.1097/MD.0000000000043440

**Published:** 2025-07-18

**Authors:** Koji Uchino, Ayane Matsuzaki, Shinzo Yamamoto, Hiroyoshi Taniguchi, Hideo Yoshida

**Affiliations:** a Department of Gastroenterology, Japanese Red Cross Medical Center, Tokyo, Japan.

**Keywords:** cancer, hepatocellular carcinoma, spontaneous regression, tumor

## Abstract

**Rationale:**

The spontaneous hepatocellular carcinoma (HCC) regression is rare. The underlying mechanism remains unclear. Elucidating this mechanism may lead to a novel HCC treatment, especially in the era of immunotherapy, because immunological reactions are among the possible mechanisms of spontaneous HCC regression.

**Patient concerns:**

A 63-year-old man fell and fractured his right olecranon. Before surgery for bone fracture, an abnormal liver function test result was noted and HCC was also suspected by ultrasonography and tumor markers. The patient was a heavy drinker and a daily smoker and was instructed to abstain from alcohol and smoking.

**Diagnoses:**

HCC was definitively diagnosed by dynamic computed tomography immediately after surgery for bone fracture. The main tumor was 6 cm in size and was accompanied by multiple small lesions.

**Interventions:**

Immediately before starting treatment for HCC, tumor markers were markedly decreased (alpha-fetoprotein, from 3098 to 361 ng/mL; des-gamma-carboxy prothrombin, from 2083 to 47 mAU/mL). Considering the possibility of spontaneous regression of the tumors, the patient was followed carefully without treatment.

**Outcomes:**

During the follow-up without treatment, the tumor markers decreased further, and the tumors disappeared from the image.

**Lessons:**

Alcohol and smoking abstinence and surgical procedures were considered possible causes for the spontaneous HCC regression.

## 1. Introduction

Spontaneous cancer regression is rare. In 1956, Everson and Cole defined spontaneous cancer regression as “the partial or complete disappearance of a malignant tumor in the absence of all treatment or in the presence of therapy, which is considered inadequate to exert a significant influence on neoplastic disease.”^[1]^

Hepatocellular carcinoma (HCC) is one of the most common malignancies and the third leading cause of cancer deaths worldwide, with a 5-year survival rate of approximately 18%.^[2]^ Johnson et al (1972) first reported the spontaneous HCC regression.^[3]^ To date, several cases of spontaneous HCC regression have been reported. Based on the analysis of previous case reports, 2 major theories that may explain spontaneous HCC regression have thus far been proposed: tumor hypoxia and systemic immunologic response.^[4,5]^ However, the exact underlying mechanism remains unclear. Elucidating this mechanism may lead to a novel HCC treatment, especially in the era of immunotherapy, because immunological reactions are among the possible mechanisms of spontaneous HCC regression. Here, we report a case of spontaneous HCC regression possibly triggered by alcohol and smoking abstinence and surgical procedures.

## 2. Case report

**Figure 1.**
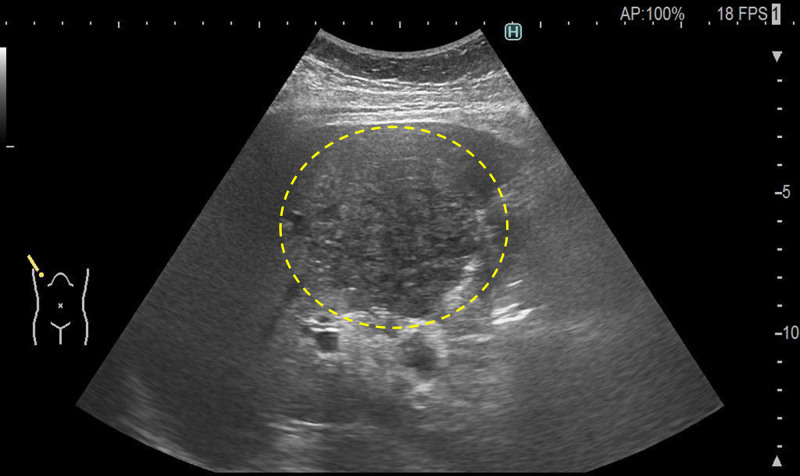
Ultrasonography showed a 6-cm hypoechoic lesion with indistinct margins in the right liver lobe.

**Figure 2.**
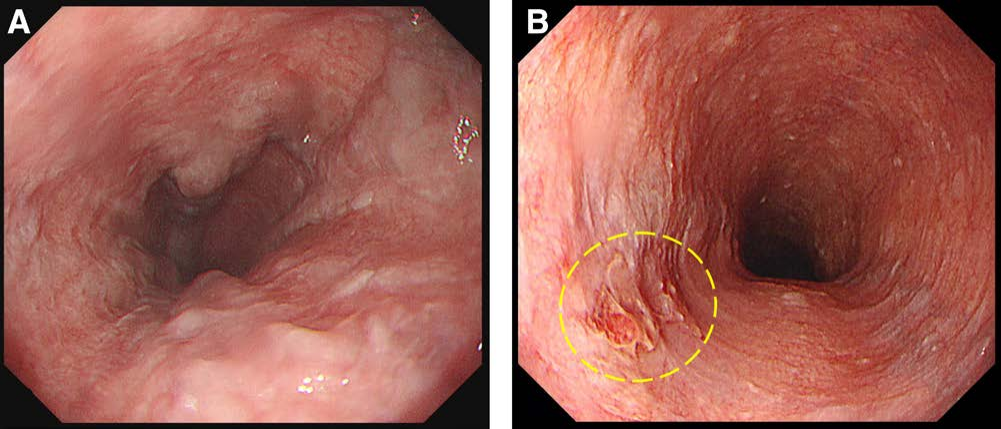
The upper gastrointestinal endoscopy revealed esophageal varices (A) and early esophageal cancer (B).

**Figure 3.**
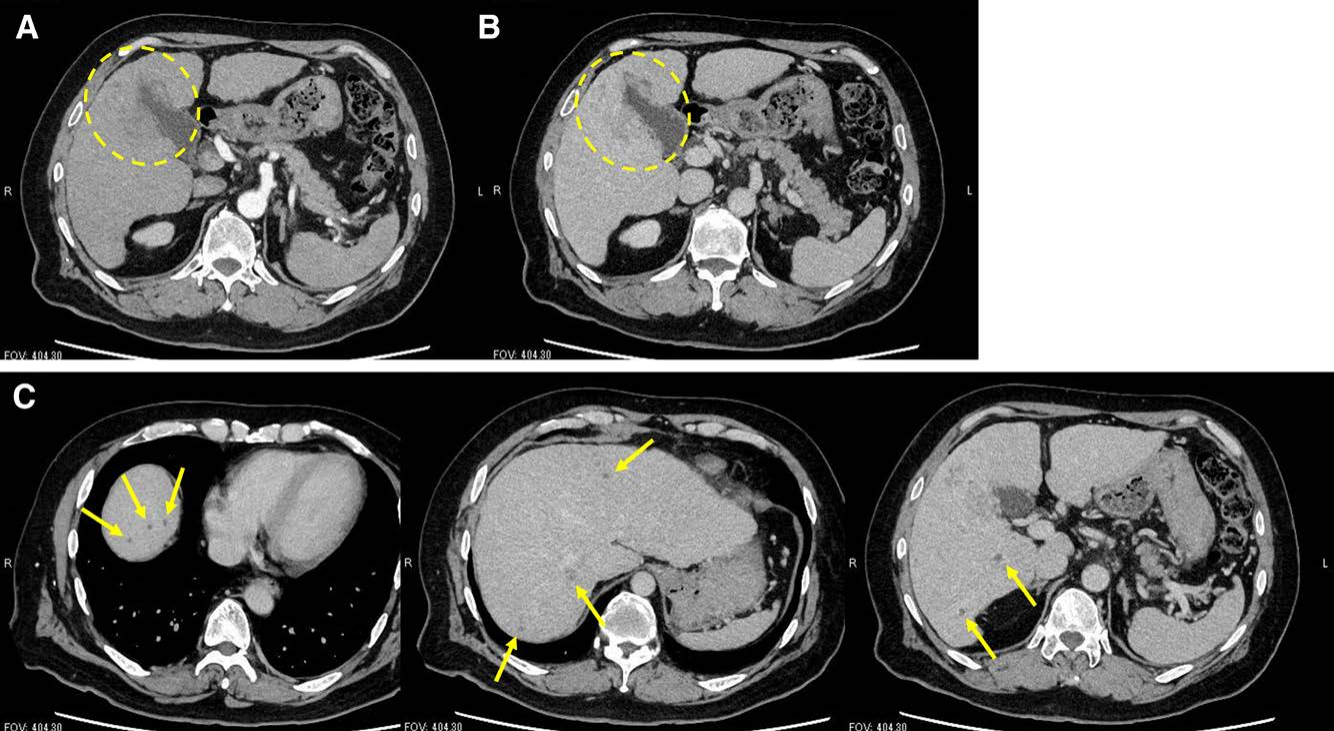
After surgery for the bone fracture, 32 d after the first visit, a dynamic computed tomography was taken. Tumor stain was not seen in the arterial phase (A). A 6-cm low-density lesion with indistinct margins in the right liver lobe in the equilibrium phase (B). Multiple hypovascular nodules similar to the main lesion were also detected (C).

A 63-year-old man fell and fractured his right olecranon. The patient was referred to our orthopedic department for surgery. He was 182 cm tall and weighed 95 kg. He had a history of alcoholic liver disease, type 2 diabetes mellitus, hypertension, reflux esophagitis, and thoracic aortic aneurysm. The patient was treated with ursodeoxycholic acid, glimepiride, azilsartan, amlodipine, enalapril, trichlormethiazide, and vonoprazan. He did not take any herbal medicines. He was a heavy drinker (110 g of alcohol per day) and had smoked 15 cigarettes per day for approximately 40 years. During preoperative screening, an abnormal liver function test result was noted. The blood test results were as follows: white blood cell count, 6620/µL; hemoglobin concentration, 11.8 g/dL; platelet count, 17.0 × 10^4^/µL; prothrombin activity, 80.0% (prothrombin time-international normalized ratio, 1.12); aspartate aminotransferase, 116 U/L; alanine aminotransferase, 37 U/L; alkaline phosphatase, 89 U/L; gamma-glutamyl transpeptidase, 392 U/L; total bilirubin, 3.0 mg/dL; albumin, 3.6 g/dL; blood urea nitrogen, 13 mg/dL; creatinine, 1.04 mg/dL; blood glucose 173 mg/dL; glycated hemoglobin A1c, 6.5%. Test results for hepatitis B surface antigen and hepatitis C antibody were negative. The serum alpha-fetoprotein (AFP) level was markedly elevated at 3098 ng/dL. Des-gamma carboxyprothrombin (DCP), a protein induced by vitamin K absence-II (PIVKA-II), was also markedly elevated at 2083 mAU/mL. Ultrasonography detected a 6-cm hypoechoic lesion with indistinct margins in the right liver lobe (Fig. 1), which was suspected to be HCC based on the AFP and DCP (PIVKA-II) elevation. Upper gastrointestinal endoscopy revealed esophageal varices (Fig. 2A), and the patient was diagnosed with alcohol-related cirrhosis. Endoscopy also detected an early esophageal cancer (Fig. 2B), and a biopsy of the esophageal lesion revealed squamous cell carcinoma. Before further evaluation of the liver lesion, surgery for the bone fracture was scheduled, and the patient was instructed to abstain from alcohol consumption and smoking. The patient was hospitalized and underwent plate osteosynthesis under general anesthesia 4 days after the initial visit. No blood transfusions were required during the perioperative period. After discharge, 32 days after the first visit, dynamic computed tomography (CT) showed a 6-cm low-density lesion with indistinct margins in the right liver lobe in the equilibrium phase. Multiple low-density nodules, similar to the main lesion, were also detected in the liver (Fig. 3). Although no tumor staining was seen in the arterial phase of dynamic CT, HCC was diagnosed considering the markedly elevated tumor markers. Macroscopic vascular invasion and extrahepatic metastases were not detected.

As the esophageal cancer was at an early stage, treatment for HCC was prioritized. Atezolizumab plus bevacizumab therapy was planned. However, the tumor markers measured immediately before starting therapy were markedly reduced (AFP, 361 ng/mL; DCP, 47 mAU/mL) (53 days after the first visit). Lectin-reactive AFP (AFP-L3) was also measured for the first time at a high level of 64.6%. No significant changes were seen on CT images. The possibility of spontaneous regression of the tumors was considered. We offered the patient the option of starting treatment as scheduled or careful observation for a while. He chose the option of careful observation. The abnormal liver function test results also tended to improve (aspartate aminotransferase, 51 U/L; alanine aminotransferase, 22 U/L; gamma glutamic transpeptidase, 125 U/L; total bilirubin, 1.7 mg/dL), probably due to alcohol abstinence.

**Figure 4.**
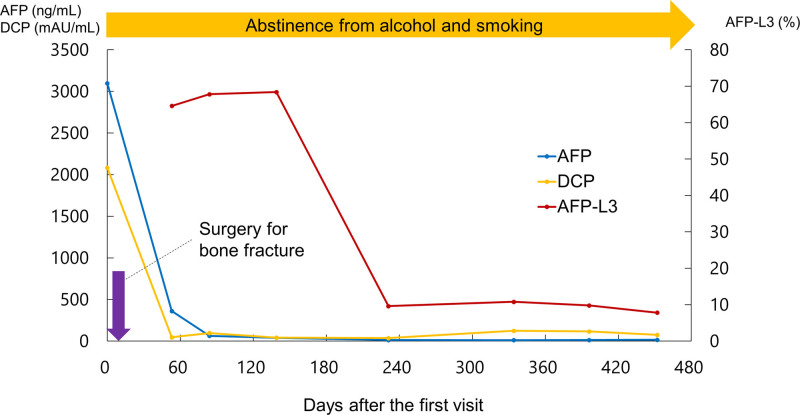
The patient was instructed to abstain from alcohol and smoking at the first visit. Four days after the first visit, surgery for the bone fracture was performed. Fifty-three days after the first visit, without treatment, the serum level of alpha-fetoprotein (AFP) and des-gamma carboxyprothrombin (DCP) markedly decreased from those at the first visit (AFP, from 3098 to 361 ng/mL; DCP, from 2083 to 47 mAU/mL). Concurrently, lectin-reactive AFP was measured for the first time with a high level of 64.6%. During the follow-up, these 3 markers decreased further and did not increase again.

**Figure 5.**
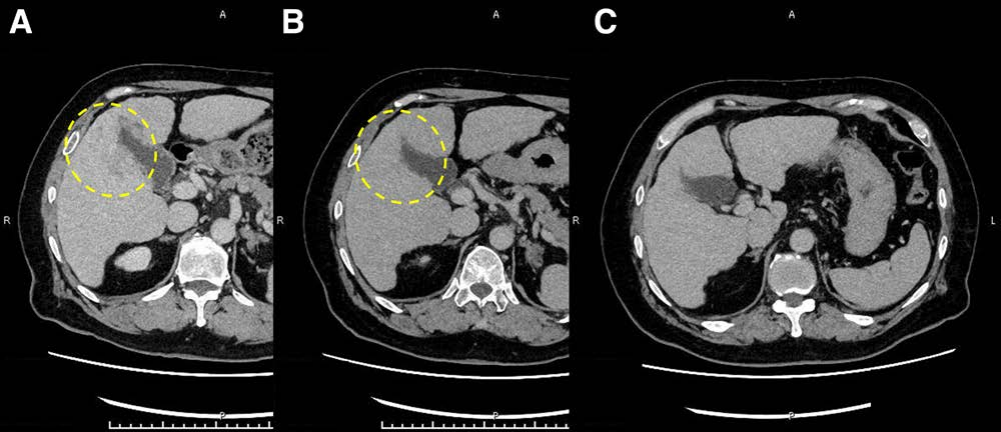
Imaging changes of the main tumor in computed tomography (equilibrium phase). (A) Image at the first diagnosis (32 days after the first visit); (B) the tumor shrank and became obscure (139 d after the first visit); (C) the tumor disappeared completely (334 d after the first visit).

During follow-up, tumor marker levels decreased further and did not increase again (Fig. 4). On the CT image, all tumors (the main and multiple small lesions) had shrunk and then regressed completely (Fig. 5). To treat the thoracic aortic aneurysm, the patient underwent thoracic endovascular aortic repair 186 days after the initial visit.

The patient underwent endoscopic submucosal dissection for early esophageal cancer 427 days after the first visit. Pathological examination of the resected specimen confirmed the diagnosis of early esophageal squamous cell carcinoma, and curative resection was performed.

No recurrence was detected in CT at 452 days after the first visit. He died suddenly at home 561 days after the initial visit. Although the cause of death is unknown, HCC was unlikely to be the cause of death as no recurrence was detected on CT 4 months before death.

## 3. Discussion

Spontaneous HCC regression is rare. Huz et al reviewed previous reports and summarized 75 cases of spontaneous HCC regression in 2012.^[4]^ Oquiñena et al reported that the rate of spontaneous partial HCC regression was 0.4%, and complete regression was extremely rare when reviewing 10 phase III clinical trials of HCC with control groups receiving no treatment or best supportive care.^[6]^

The mechanism underlying spontaneous HCC regression remains unclear. Several studies have categorized the factors involved in spontaneous regression into 2 major types: (i) tumor hypoxia and (ii) systemic immunological reactions.^[5]^ Factors categorized as tumor hypoxia include tumor thrombosis of the hepatic artery or portal vein, hepatic angiography, rapid tumor growth, hepatic arterioportal shunts, massive gastrointestinal hemorrhage, hemodialysis, and surgical procedures. Factors categorized as immunological reactions included alcohol or smoking abstinence, herbal medicines, prolonged fever, antidiabetics, and vitamin K administration. Among these factors, abstinence from alcohol^[7–12]^ and smoking^[8,11,13,14]^ and surgical procedures^[15–17]^ may have contributed to the spontaneous regression in this case.

Alcohol abstinence is one of the most frequently reported factors associated with the spontaneous HCC regression. In this case, the patient abstained from alcohol consumption and smoking. Similarly, Grossmann et al and Saito et al reported spontaneous HCC regression after alcohol and smoking abstinence.^[8,11]^

Sato et al reported a case of spontaneous regression following surgery for a bone fracture.^[15]^ In their case, the patient received a blood transfusion during the perioperative period. They suggested that the blood transfusion and surgical procedure might have contributed to the spontaneous regression. However, the patient did not receive a blood transfusion in this case. It is conceivable that the surgical procedure itself may have contributed to spontaneous regression in this case.

Tumor thrombosis of portal vein is one of the well-known possible causes of spontaneous HCC regression through tumor hypoxia.^[14,18–21]^ In our case, portal vein tumor thrombosis was not radiologically detected.

Recently, several cases of spontaneous HCC regression after COVID-19 infection and/or vaccination were reported.^[16,22]^ The patient in our case was not infected with COVID-19 prior to the spontaneous tumor regression. We could not obtain a history of COVID-19 vaccination from him.

Despite various clinical case reports of spontaneous HCC regression, few studies have attempted to elucidate the mechanism of spontaneous tumor regression from an immunological perspective. The role of the immune environment has been implicated with certain cytokines and inflammatory markers,^[23]^ such as IL-18,^[24]^ TNF-α^[25]^ and IL-2, IL-6 and IFN-γ^[26]^ identified in some case reports. Furthermore, Arjunan et al demonstrated a higher propotion of activated NK cells and a highly pro-inflammatory and cytotoxic T cell response in ex vivo stimulation measured by expression of TNF-α, IFN-γ and granzyme B.^[27]^

Interestingly, although the HCC regressed spontaneously, the comorbid early esophageal cancer persisted in this case. Grossmann et al have reported a similar case.^[8]^ In their case, although HCC regressed after alcohol consumption and smoking abstinence, early gastric cancer persisted. The tendency for spontaneous regression may be related to cancer type.

One limitation of this case report is that HCC was not pathologically diagnosed despite atypical imaging findings. In this case, the tumors were not hypervascular. However, the extremely high levels of HCC tumor markers (i.e., AFP, AFP-L3, and DCP) supported the clinical diagnosis. Moreover, the disappearance of the lesions on CT, along with a decrease in tumor markers, also suggested that the lesions were HCC. Another limitation is the difficulty in determining which of the several possible causes contributed most to the spontaneous regression. A third limitation is the lack of pathological proof of complete disappearance of the tumors, because no postmortem autopsy was performed.

In summary, we present a case of spontaneous HCC regression which may be triggered by alcohol and smoking abstinence and surgical procedures. From our and other previously reported cases, it is speculated that rapid and dynamic changes in the immune environment mediated by certain cytokines are responsible for the spontaneous HCC regression. However, exact mechanism is still unclear. Further study is needed to develop novel immunotherapies in HCC.

## Acknowledgments

We would like to thank Prof. Yosuke Tsuji (Department of Gastroenterology, Graduate School of Medicine, The University of Tokyo) for performing endoscopic submucosal dissection for esophageal cancer and Editage (www.editage.jp) for English language editing.

## Author contributions

**Conceptualization:** Koji Uchino.

**Data curation:** Koji Uchino.

**Investigation:** Koji Uchino.

**Methodology:** Koji Uchino.

**Writing – original draft:** Koji Uchino.

**Writing – review & editing:** Koji Uchino, Ayane Matsuzaki, Shinzo Yamamoto, Hiroyoshi Taniguchi, Hideo Yoshida.
